# A Qualitative Exploration into the Sensory Experiences of Autistic Mothers

**DOI:** 10.1007/s10803-021-05188-1

**Published:** 2021-07-12

**Authors:** Moyna Catherine Talcer, Orla Duffy, Katy Pedlow

**Affiliations:** 1 Moyna Talcer LTD, Occupational Therapist, PO Box 378, Wallington, SM6 6DQ Surrey UK; 2 Centre for Health and Rehabilitation Technologies, Institute of Nursing and Health Research, Ulster University, Shore Road, Newtownabbey, BT37 0QB UK; 3 Centre for Health and Rehabilitation Technologies, Institute of Nursing and Health Research, Ulster University, Shore Road, Newtownabbey , BT37 0QB UK

**Keywords:** Autistic mothers, Experience, Female autism, Motherhood, Sensory, Thematic analysis

## Abstract

Research has found 96% of autistic individuals experience sensory processing difficulties, and being a parent presents many sensory demands that may be especially challenging for autistic mothers. Despite the high prevalence, no research exists exploring the sensory experiences of autistic mothers, highlighting the gap in current knowledge. Semi-structured interviews were conducted with 7 autistic mothers, data were analysed using thematic analysis identifying 5 major themes: antenatal experiences, sensory experiences in motherhood, the impact of sensory processing difficulties, strategies and needs, diagnosis. This research provides greater insight and understanding into the sensory experiences of autistic mothers which can influence earlier diagnosis and inform appropriate support and adaptations for autistic mothers in a variety of different sectors and highlights a possible emerging role for Occupational Therapists.

## Introduction

Autism Spectrum Conditions (ASC) is the preferential term for Autism Spectrum Disorder (ASD), within the Autistic community as it is less stigmatising (Dudas et al., 2017). It is a lifelong, neurodevelopmental condition presenting as difficulties with social communication and interaction, restricted and repetitive patterns of behaviours, activities, or interests. These are present from childhood however may not manifest until social demands outweigh the person’s capabilities, and limit and impair everyday functioning. Atypical responses to sensory stimuli have long been associated with the autistic population, correlating highly with functional impairments and higher levels of distress. The American Psychiatric Association’s revision of the Diagnostic and Statistical Manual included sensory over and under reactivity within the diagnostic criteria for ASC (DSM-5 2013).

Sensory-motor and perceptual difficulties within the autistic population were first described by Wing (1969) who noted that behaviours of autistic children differed from those in neurotypical children and those with other developmental difficulties. Early autism research was heavily focused on the paediatric population and as a result, health and education provisions and resources for children were well established. As advances are made within the fields of neuroscience, and greater awareness and understanding is growing, ASC is now recognised as a lifelong condition, however, service provision for adults with ASC is still within its infancy (Murphy et al., 2016).

Most research regarding sensory processing within autistic populations comes from the field of paediatrics. However autistic children usually grow into autistic adults and sensory processing difficulties are known to persist into adulthood (Ben-Sasson et al., 2010; Goldsmith et al., 2007), where links have been made with increased stress levels (Cesaroni & Garber, 1991; Smith & Sharp, 2013; Horder et al., 2014). Despite this, very little research into sensory processing has been carried out with autistic adults, and even less with female autistic adults, highlighting a gap in the current knowledge base.

Sensory Integration was defined as "*the neurological process that organizes sensation from one's own body and from the environment and makes it possible to use the body effectively within the environment*" Ayres (1972a, p. 11). Sensory over and under reactivity is also referred to as sensory modulation, where patterns of sensory integration dysfunction include over, under or fluctuating responses to sensory stimuli (Boyd et al., 2010; Fisher et al., 1991; Greenspan & Wieder, 1997). Poor sensory modulation is thought to present as both behavioural and emotional regulation difficulties, including distractibility, impulsivity, increased activity level, disorganisation, anxiety, poor emotional and self-regulation control and can lead to difficulties engaging in the activities of daily living (Ayres, 1972a, 1972b; Cohn et al. 1999; Bundy et al., 2002). Behavioural indicators for sensory over-reactivity can include, but are not limited to, hypersensitivity or an over response to unexpected sounds, bright and contrasting lights, strong smells, aversions to specific textures, difficulty receiving light touch, over-reactivity to changes in temperature and pain and anxiety reactions when an individual’s gravity is challenged. Underactivity behaviours are thought to be more difficult to research due to the behavioural responses being less clear and obvious (Lane, 2019).

In the UK, the prevalence of diagnosed autistic adults (diagnosed as an adult) is evaluated at 1.1% (Brugha et al., 2011) with the estimated ratio of male to female being 4:1. ASC diagnostic assessment tools have been based upon predominantly male samples; therefore the behavioural and diagnostic criteria are heavily biased towards males, leading to increased difficulty in recognising female autistics, creating a delay in diagnosis (Begeer et al., 2012; Devukot et al., 2017; Dworzynski et al., 2012; Giarelli et al., 2010; Kreiser & White, 2014; Russell et al., 2011, Thompson et al., 2003). Emerging research is finding that autistic women have behavioural differences in the way they express their autistic symptoms, (Hull et al., 2017, 2020), and are significantly at higher risk compared to males, of being misdiagnosed with mental health difficulties or left undiagnosed (Attwood, 2007; Constantino, 2011; Goldman et al., 2013; Kreiser & White, 2014; Lai & Baron-Cohen, 2015). Evidence suggests that early diagnosis and intervention is imperative in the long-term trajectories, adaptive behaviours and quality of life of autistic individuals (Elder, 2017).

The role of a parent is a stressful one, however, when a diagnosis of ASC is present, additional challenges exist*.* The sensory demands of parenthood, therefore, may be especially challenging for autistic mothers. However, at present little research exists, exploring the sensory behaviours and experiences of autistic females (Gould, 2017; Leedham et al. 2020). The work of Behrman and Butler (2007) and Moutquin (2003) found that autistic mothers were at an increased risk of having a pre-term birth due to increased levels of stress. This has been supported by others, suggesting that autistic mothers have potential risk factors associated with sensory processing difficulties, including but not limited to, delivery complications, maternal stress and illness and maternal use of medications (Ben-Sasson et al., 2009; Keuler et al., 2011; May-Benson et al., 2009; Schneider et al., 2007).

Autistic mothers experienced higher stress responses due to heightened sensitivity to sensory stimuli, causing difficulties adapting to the experiences of motherhood (Sundelin et al., 2018; Gardner et al., 2016; Rogers et al. 2016). This included heightened sensory perception to tactile, visual, and auditory input which impacted other areas of their lives including intimate relationships, parenting styles and comfort with accessing health services, however, these challenges were not further explored. An online survey (Pohl et al., 2016) completed by 325 autistic mothers reported that autistic mothers also felt isolated and misunderstood by professionals and experienced greater levels of anxiety, suggesting that autistic mothers are within a higher-risk population. Studies to date have only focused on the early experiences of motherhood including the prenatal and perinatal periods, highlighting a lack of research focusing on the postnatal and the child-rearing stages. None of these studies has specifically investigated sensory experiences. Therefore it is unknown if the reported risks factors continue into the child-rearing period.

## Aims and Objectives

This study aimed to explore the sensory experiences of autistic mothers and investigate if those sensory experiences have any impact on the participant’s role as a mother and what the consequences of this may be. This study, therefore, aims to (1) explore the sensory experiences of autistic mothers; (2) To identify how sensory experiences impact the participant's role as a mother; (3) What strategies have helped manage the sensory experiences.

## Methods

A Qualitative methodology with semi-structured interviews conducted via telephone was used to understand the sensory experiences of autistic mothers. Treweek et al. (2019) discussed the importance of research ascertaining how autistic people feel. Interviews provide a detailed investigation of the lived sensory experiences to explain and explore delicate phenomena, thereby offering clarification and a deep understanding of the phenomena being explored (Ritchie, 2003). This method is extremely well suited to in-depth studies with a smaller number of participants, where the inquiry is based on careful examination of the human experience (Smith et al., 2009).

### Participants

Study participants were selected using volunteer sampling and recruited after responding to the study flyer which was posted on social media platforms and the Cambridge Autism Research Database (CARD). Interested participants were sent a Participant information form with further details about the study if they wished to participate in the study and after successfully meeting the eligibility criteria, they provided informed consent and were sent details of their interview. Participant characteristics were collected to include age, ethnicity, source of autism diagnosis, any comorbidities, number of children, whether any children were autistic (Table 1). None of the participants was previously known to the research team.

**Table 1:** Participant characteristics

Participant ID	Age range	Ethnicity	Diagnosed by	Other conditions	Children	Children diagnosis
P1	35–40	White	Psychiatric Nurse	Anxiety	2 aged between 6 and 7	1 ASC
P2	50–55	White	Doctor	NA	2 aged between 17 and 22	NA
P3	30–35	White	Neurodevelopmental OT	Dyslexia and Dyspraxia	2 aged between 2 and 7	NA
P4	40–45	White	Consultant Psychiatrist	NA	1 aged 9	ASC
P5	35–40	NA	Psychiatrist	OCD, Anxiety, IBS, Migraines	3 aged 9–21	1 ASC
P6	50–55	White	Consultant Psychiatrist	NA	2 ages 16–18	1 ASC
P7	45–50	White	Psychiatrist	Depression	2 aged 10–12	2 ASC

Inclusion criteria: (1) identify as female, (2) diagnosed as autistic by a professional, (3) living with their child, (4) living in the UK, (5) access to the internet or willing to use the telephone, (6) speak English and (7) over the age of 18.

Exclusion criterion: (1) known significant mental health illnesses i.e. known significant mental health illness including Bipolar Disorder, Schizophrenia and psychosis, (2) Physical disability which limits mobility or causes pain, (3) known brain injury or cognitive impairment.

### Interview Questions

The researcher developed an interview guide with six open-ended questions and suggested probes following Patient and Public Involvement (PPI). (See Appendix 1). Questions were developed and informed by the literature which identified that autistic mothers often experienced an increase in their sensory experiences following pregnancy and the birth of their child impacting their engagement in occupational roles as a result (Gardner et al., 2016). Questions were also informed following online consultation within closed online groups with autistic mothers. Therefore, questions were developed to include pre and post-birth sensory experiences and questions regarding participation in the activities of daily living.

### Pilot Interview

A pilot telephone interview was conducted with the first participant recruited to the study where they were invited to provide feedback on the participant information forms, consent forms and recruitment process and the appropriateness of the interview topic guide. This was to ensure participants were able to give full and coherent accounts of the central issues being explored. Following the pilot interview, the topic guide and research procedures were deemed fit for purpose and no changes were required, therefore the pilot interview data was included in the total data set and analysis process.

### Data Collection

Data was collected between May 2019–June 2019. All participants were informed of the research aims and signed consent forms. All interviews were conducted in a quiet and confidential room by the lead researcher who is an experienced qualified Occupational Therapist and Advanced Practitioner in Sensory Integration. No other people were present during data collection. All participants were made aware of the researcher’s insider researcher status before the interview. Interviews lasted on average 51 min (range 38–67 min) where the researcher made comprehensive field notes. The interviews followed a thematic script prepared by the lead researcher that was informed by literature and previous knowledge (Appendix 1).

Interview audio recordings were saved to a password-protected file and computer immediately after the interview and original recordings were deleted from external devices. Interviews were transcribed verbatim using a GDPR compliant transcription service, checked for authenticity against the original recording, then sent to the participant with all identifiable data being assigned a code to ensure anonymity as a record of the interview and to ensure rigour.

### Data Analysis

A six-staged theoretical thematic analysis framework was applied as recommended by Braun and Clarke (2006) using deductive analysis with an overarching lens of Sensory Processing Difficulties. The researcher read and re-read the transcriptions sequentially, making initial notes in the margins of the transcripts. Next, codes were generated relating to the research questions, alongside interesting features of the data, then these codes were analysed for themes. Data was gathered sequentially from each interview and themes were generated. Subsequent interview data either added to the existing themes or new themes were developed until no new themes were found, at which point saturation was reached. Each participant was invited to comment on the summary of themes in their interview and add any thoughts via email. None of the participants suggested any changes or amendments and of the two participants who sent comments, they agreed that the themes accurately described their experiences. Next, a thematic map was created relating to the research questions, and themes reviewed and refined to form an overall analysis. Qualitative research good practice guidelines (Mays & Pope, 2000) were adhered to where the researcher carried out ongoing credibility checks to ensure that data was analysed appropriately, including regular checking and consensus of main interpretation and themes generation with the researchers two supervisors along with member checking of the themes in each interview to ensure that the developed themes, accurately reflected the participant's experiences using appropriate semantic descriptions. Once a shared consensus was reached, vivid extracts were selected to relate to each theme and ensure this related to the research question before compiling a report of the analysis. Following analysis and confirmation of themes, participants were sent a copy of the final analysis of the completed study. The researcher kept a reflective journal throughout, and potential bias was considered, identified and documented by the principal researcher via bracketing. The researcher’s analysis process was reviewed by the research team members and checked for reliability throughout.

### Ethical Considerations

Ethical approval was sought from Ulster University Research Ethics Committee (UREC). All identifiable data was processed in line with GDPR requirements and coded to ensure anonymity. Codes were stored separately, and all other data was password protected and stored on a computer with a secure password known only to the researcher. Confidentiality was maintained throughout the study. Participants provided informed consent to participate. No participants withdrew consent.

The researcher created two protocols and policies to adhere to throughout the research process including, a Distress Policy and a Vulnerable adult’s policy and sent all participants a debrief document following their participation signposting to services offering support following their participation.

The researcher is an insider researcher, meaning that she is studying a group of people of which she too is a member. She, therefore, played two main roles, the researcher and the researched (Greene, 2014). To reduce potential bias, the researcher adhered to a trustworthiness criterion in all areas of the research (Lincoln & Guba, 1985) carrying out several processes to reduce bias and improve trustworthiness in all aspects of the study. This included prolonged engagement with the data, peer debriefing with her supervisors, member checking by inviting participants to review and comment on the summary of themes derived from their transcripts, and the data analysis to confirm meaning, disciplined bracketing of assumptions and pre-existing beliefs and experiences, making detailed reflections paying close attention to personal bias and views and holding a commitment to accurately and adequately representing the participants experiences through member checking.

## Results

Eight autistic mothers applied to take part in the study. Seven met the eligibility criteria. One mother was under the age of 18 years and was therefore excluded. Table 1 shows the following characteristics of the participants; age, ethnicity, source of diagnosis of autism, any comorbidities, number of children, whether any children were autistic.

### Qualitative Analysis

**Fig. 1:**
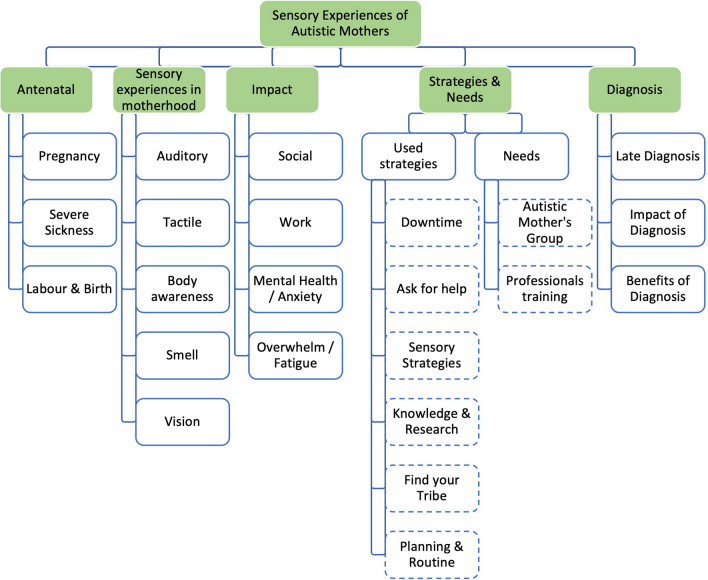
Themes and subthemes

From the rich data set gathered, 5 overarching themes were generated with 17 subthemes (Fig. 1) which are presented in Fig. 1. No adverse effects were reported by participants and all interviews were completed as intended.

### Theme 1: Antenatal

This theme captures the challenges experienced by the participants throughout the pregnancy period and highlights the high prevalence of severe sickness in pregnancy.

#### Sub-theme 1.1: Pregnancy

All participants reported that pregnancy made their sensory experiences noticeably more heightened, and for some, this never really decreased to baseline following the birth of their child. For most, the internal sensations of being pregnant were very difficult to cope with.

“When he (baby) started moving inside me it was unbearable. It was awful because he used to have hiccoughs quite a lot, I mean you do put up with it but it was just awful. I used to describe it as having an alien in me”. (P4).

In addition, the mothers had many medical appointments during pregnancy which was difficult both on a social and sensory level.

“My pregnancies were unpleasant, and it could have been made so much simpler and easier for me if they (medical professionals) could have made little changes, it would make a massive difference, especially for an autistic mother”. (P5).

#### Sub-theme 1.2: Severe Sickness

Most of the participants experienced severe and prolonged sickness throughout their pregnancy. Some reported that their sickness felt “different” when they compared their experiences to other neurotypical mothers, and it was difficult to express to others how severe their sickness was as others were unable to detect the sensory stimuli as intensely as they did.

“I think my sensory problem is I can’t process too much visual information. It makes me feel overwhelmed and anxious. And in pregnancy… I noticed I was different from others. The visual stuff, staring at my computer screen would make me feel incredibly sick and it was getting worse and worse over the days. (P1).

#### Sub-theme 1.3: Labour and Birth

All participants were undiagnosed at the time of childbirth; however, some anticipated sensory aspects of the labour and birth would be challenging and so were able to successfully make requests for small sensory accommodations during their labour process.

“Look, I know this sounds weird but I need that (labour monitor) on my ankle instead.”. They did find it strange…I obviously understand now it’s a sensory issue. I said, “It’s gonna really stress me out otherwise.” It’s gonna be painful. It was too much. The touch was too much”. (P3).

Other participants did not feel listened to by medical professionals and found it extremely challenging throughout the labour process to verbalise and explain what they were experiencing. Participants did not feel the medical staff could understand nor meet their requests to adapt to the physical sensory environment leading to insufficient pain relief and inadequate care management for some.

“It (labour) was difficult especially when you are having to be touched by medical people, I don’t like being touched at all by anybody, not even my kids, sometimes not even my partner, it hurt. It’s hard to explain but it hurt… It was horrific, it was really horrific because you’re having people coming in all the time in the room, the lights are really bright, it’s noisy, what affected me massively was I could hear, because my hearing is so clear I could hear women from far away in labour and it affects you, but trying to explain that to other people they just don’t get it, they don’t understand” (P5).

Other participants expressed that the predictability and formulaic nature of a planned C-section along with access to familiar staff was very reassuring during the labour and birth process.

“It (c-section birth) was fine because it’s all very formulaic, so you know what’s going to happen and they tell you what they’re doing. So, the actual delivery wasn’t a problem but again afterwards, many, many issues!”. (P4).

### Theme 2: Sensory Experiences in Motherhood

This theme describes the sensory experiences of autistic mothers in the post-natal period and beyond.

#### Sub-theme 2.1: Auditory

Auditory overactivity was reported by almost all of the participants as one of the main sensory areas that caused increased stress and anxiety in motherhood, impacting mental health.

“(Baby crying) it was like ice down my back, I couldn’t cope with it whatsoever, so I’d go to her instantly to stop it. Yeah, so it was, it was just awful”. (P7)

However, the auditory challenges changed as their children grew and developed speech.

“It’s just exhausting because he’s actually speaking to me, you know. I have to tolerate quite a lot of it…Yeah, for several hours if left unchecked (child talking a lot), it’s excruciating. I want to shrivel” (P7).

Most participants commented that the toys children play with are overwhelming from a sensory perspective.

“As they get older you’ve got all these brightly coloured, noisy, jangly, interactive, horrible stuff like, like you know, you press a button and bang!… The banging…! You know, so it is very overwhelming” (P4)

#### Sub-theme 2.2: Tactile

The role of a mother naturally involves a high degree of tactile input. For the majority of participants, the sudden influx of tactile stimuli became completely overwhelming.

“My body was constantly and still is invaded and I find that so difficult. I feel like my soul is itchy. It’s difficult isn’t it because I love him dearly and, you know, I know he’s showing me affection, but I cannot, it’s like I’m too full, it’s a painful fullness and my skin’s going to burst, it’s awful.” (P7).

Breastfeeding was highlighted as being very painful and overwhelming, placing a significant demand on their tactile system, resulting in becoming easily overwhelmed and impacting other relationships within the family. Yet most participants expressed how they tried their best to support their infant to breastfeed even though it was at times excruciating for them, as they knew that was the best support for their child.

“I breastfed for two years and I got very touched out, to the point where I couldn’t stand my partner touching my arm, that contact can get very overwhelming very quickly. It’s got to the point now where that’s painful. I think the skin has become so over-sensitized that it’s painful (P3).

#### Sub-theme 2.3: Body Awareness

Following the birth of their child, some participants reported their body awareness was altered, compromising their recovery from childbirth and subsequent transition into motherhood.

“I had some complications after the birth…. Cos I’m not overly aware of my body, I’ve moved too quickly up the bed, and I accidentally tore my stitches and they got infected”. (P3)

### Theme 3: Impact

The impact of increased sensory input upon the autistic mothers as they reared their children affected them within several different environments and activities.

#### Sub-theme 3.1: Social

Before becoming mothers, most of the participants would avoid social situations and seek time alone as a way of managing sensory overwhelm. However, during the early developmental phase, and as their children started accessing education, there was an increased need for participating in regular external play and social activities. Most participants reported group activities were too overwhelming.

“The problems started when I had to take them to playgrounds, groups, baby groups, and also, that’s when, you know, I really start to struggle with too much going on around me and I can’t really carry a conversation. It makes me feel quite like a failure. You know, mothers, they invite me once for a coffee and they don’t invite me a second time and it’s upsetting. I’ve been upset ever since”. (P1).

Most participants reported feeling guilty that they were unable to engage in their motherly duties in the same way as other neurotypical mothers, leading to them feeling their child was missing out on key experiences.

“I would say my impact as a mother is massively affected because if I go to a parent's evening, the noise and the light. It’s just too overwhelming and I ended up having a meltdown. So, my kids then have to not have me go to their parents evening because of how I am, so I feel bad.” (P5).

#### Sub-theme 3.2: Work

Work was an important role for most participants, however, due to the increased sensory demands of being a mother, work was not always sustainable.

“I can’t work because I’m full of kids, of parenting, I can’t do anything else unfortunately even though I don’t have them for six hours of the day(going to school), you know.” (P7).

Some participants reported that their employer lacked understanding of their specific sensory needs within the workplace.

“I rang up my boss to have a day off sick a couple of months ago cos I was having panic attacks. “Well, have you rung your doctor?” I’m like, “I don’t need to ring my doctor.” Like, tablets aren’t gonna help this…. I’m overwhelmed!”. (P3).

#### Sub-theme 3.3: Mental Health/Anxiety

Most participants reported increased levels of anxiety upon becoming a mother, linking this to the inability to revert to previously used coping strategies of withdrawal and being alone to recharge and reduce sensory overwhelm. In addition, some of the mothers experienced a delay in developing an emotional connection to their baby when compared to other neurotypical mothers.

“I feel I identify as having perhaps had postnatal depression, but I don’t know whether it was that or just a meltdown really, a prolonged meltdown due to my life. Like previous coping strategies, you can’t go back to what you need”. (P4).

#### Sub-theme 3.4: Overwhelm/Fatigue

Fatigue and constant overwhelm was a major factor in participants reported experience. Yet many found they were unable to implement coping strategies as they were just so exhausted.

“I can always try harder to find ways to cope but like at the end of the day if I’m already tired, I genuinely…I haven’t got the energy to find a way to cope… I haven’t got the energy” (P2)

Some reported having to think about the needs of their child all the time contributed to their overwhelm.

“Just having this extra person, I didn’t know what to do with him, should I hold him? Should I put him down? things like nappies and… You know, I was quite freaked out … it was so overwhelming… (P4)

### Theme 4: Strategies and Needs

This theme explores the strategies participants actively employed to reduce sensory overwhelm and also identifies strategies that were desired but not implemented resulting in the identification of needs.

#### Sub-theme 4.1: Used Strategies

##### Downtime

All participants identified the need for time alone to recharge and manage their sensory needs as a mother. The downtime was essential yet not always achievable or valued by others. Most participants reported if they were unable to access downtime, they would avoid situations as a means of reducing sensory overwhelm.

“I live nearby the office; I went back at lunchtime. I had a cup of coffee and went down for a 20-min nap and it helped me. It still does. But I wish people accepted that you need that, as opposed to calling you lazy and that nobody sleeps during the day”. (P1).

For some, as their children grew older, they had more available downtime.

“Now (the children are a bit older) I can do my own thing a bit more. I can plan my day where I know I can finish (tasks) while the children are in bed. And I can do something I want to do that evening. I quite enjoy cooking and baking. It relaxes me” (P1).

##### Ask for Help

Participants generally reported finding it hard to ask for help, yet, found it invaluable when they did.

Participants reported having another person who understood their specific needs was important for them; some found it difficult to find the right kind of help and were apprehensive through fear of judgement about their ability to parent. Others were concerned if they asked for help from a medical professional, they were more likely to be at risk of receiving a misdiagnosis of a mental illness.

“Yeah, but for many, many years I needed support, it is difficult to find people to say these things to because you fear that if you speak to the GP or whoever, or the midwife they’re immediately going to go, “Postnatal depression”, (and think you are a) risk for this family or child or whatever.” (P4).

Most participants felt asking for help from other autistic mothers was of huge benefit as they did not feel judged and experienced a shared understanding.

“I would say try and get in touch (with other autistic mothers), that’s difficult in itself. So you can also share experiences because everybody’s got their own different coping strategies”. (P5)

Formal support was only accessed by one participant, and the participant reported they had to “teach” the carer how to support her sensory needs, due to lack of autism-specific training and awareness.

“I would have support for going to a hospital appointments because the hospitals have got smells galore, (but) I’m learning (teaching) her, I’m telling her (carer) what to do.”.(P5)

##### Sensory Strategies

Most participants were resourceful in finding their own sensory strategies and small adaptations were deemed to be highly effective in reducing the negative effects of sensory overwhelm. Environmental adaptations and visual strategies helped improve wellbeing. When at home, some participants used their own sensory strategies to support the regulation of their children with positive effects.

“When they were very young, they did have to listen to the same songs everywhere. On a loop. But obviously, it was children’s songs to calm them down, a lot of the time I would use music to calm them down because it calms me down”. (P2).

“I keep everything nice (and tidy) to soothe my sensory things” (P1)

Whilst others expressed the need for low lighting.

“I don’t have the big light on, I’ve got lamps because I can’t stand the light, just making the small little changes what can make it easier for the person themselves, what you wouldn’t even realise” (P5)

Others noted that whole-body stretching and physical exercise helped maintain relaxation.

“I ended up carrying him (child) around all the time as an extension to me, so instead of him being a bump in me, he became a bump on me.” (P4).

##### Knowledge and Research

Most of the participants read a lot of literature and research to help them prepare for their role as a mother, and for some, the mothering role did not come naturally. Some indirectly accessed formal education about sensory processing difficulties through their own child’s care pathway which was reported to have been very helpful. However most found that there was little relatable literature available, leading them to feel even more isolated in their experiences.

“It (reading parenting books) used to make me feel, you know, they talk about that rush of love and all that sort of stuff, and I just used to think, “I feel awful.” So, yeah, trying to access anything that let me know that this was okay to feel like this, well it wasn’t possible”. (P4).

##### Finding Your Tribe

Most participants reported making links with other autistic mothers online was of great benefit as they could be open about how they were feeling, not feel judged and develop a positive identity as an autistic mother.

“Things were very, very, very tough and thankfully I came across an online parenting forum which was largely frequented by – as it turns out many of us autistic women, and many of us had arrived there saying, “Why am I such a bad mother,” you know, “what on earth am I doing wrong?” So I found a lot of online solidarity”. (P4).

Some of the participants were able to develop meaningful friendships in person by accessing the online platforms.

“Many of the ladies who I get on very well with on this parenting forum, one of whom actually lives quite nearby, there are people that I can lean on and say, “This is happening and it’s driving me mad!” and they go, “God, I know exactly what you mean!” It’s so, so good…” (P4).

### Sub-theme 4.2: Needs

Most participants identified a clear need for networking with other autistic mothers and access to better autism awareness within statutory and medical services. Specifically, most participants felt less able to ask for help, as those in a position of authority (medical professionals) had little autism-specific training and as a result, were more likely to judge their ability to mother. Participants, therefore, highlighted the need for better training about the needs of autistic mothers.

#### Autistic Mothers’ Groups

Most participants wanted to access face to face groups for local autistic mothers, yet reported it was very difficult to find other autistic mothers.

“Just somewhere where you can meet other high functioning women who, you know, who don’t expect you to keep with their 100 WhatsApp messages a day or, you know, understand that if you message them and say, “Look, I’ve had a meltdown this morning,” (P3).

#### Professional Training Needs

Participants highlighted the need for more awareness about the sensory experiences of autistic mothers so services could provide better individualised care and make appropriate adaptations.

“Raising awareness amongst health visitors or midwives or indeed autism service for adults which are, you know, again non-existent. I would also be wanting perhaps midwives to have some sort of understanding of the impact on mothers of being pregnant if you’ve got autism, if you got sensory processing difficulties” (P4).

### Theme 5: Diagnosis

This theme highlighted the high rate of late diagnosis for autistic women and the impact of this on their overall wellbeing and development of self-concept.

### Sub-theme 5.1: Late Diagnosis

All participants reported getting their Autism diagnosis after becoming a mother. Some received their diagnosis after returning to work following the birth of their child, finding they had less energy to cope compared to pre-pregnancy, whilst others received their diagnosis following the assessment of their child.

“Going through the diagnostic procedure for him (child)..you go through it (the assessment) and you’d look at it and you’d think, “Well, that’s normal, that’s not autism, that’s what life is like.” Then you start realising, “Hang on a minute that was me. If this is my normal, then I’m autistic!” (P4).

### Sub-theme 5.2: Impact of not Being Diagnosed

Most participants reported feeling weird or different before getting their diagnosis, leading to the assimilation of a negative self-concept with many reporting a negative impact on their mental health as a result of being undiagnosed in adulthood.

“I didn’t know I was autistic, I just thought I was like a crazy person” (P2)

Some participants felt that being diagnosed later in life has affected the choices they have made in their lives.

“Autism can cloud your decision making, well it has mine and I think I honestly believe that if I was diagnosed when I was younger I probably, it’s going to sound harsh but I probably wouldn’t have had kids” (P5)

### Sub-theme 5.3: Benefits of Diagnosis

All the participants felt that a diagnosis was a powerful and important step in understanding why they found certain situations difficult, led to greater self-acceptance and allowed them to develop new coping strategies. Therefore, they were able to reduce the negative effect on her wellbeing and upon their role as a mother.

“It (diagnosis) made me feel like I’m no longer a failure as a human being, I am actually a successful person with autism, and knowing that you have got sensory issues rather than thinking there’s something wrong with you. (P4).

Participants stopped trying to fit into the “neurotypical” mould and embraced their sensory differences resulting in feeling able to be more open about their difficulties. For some, understanding their sensory needs, enabled them to drop their masking behaviours however, this required a period of adjustment to find out who they were without the mask.

“When you get a diagnosis, you go through a (grieving) process, and I think, at the moment, I’m still trying to learn what I can drop and what I need to keep” (P3)

## Discussion

Most mothers can experience stress and anxiety at some point during motherhood, however, autistic mothers with sensory processing difficulties presented with more extreme and pervasive challenges, affecting their role as a mother. It is reasonable to suggest that the sensory processing challenges, exacerbated their levels of stress and anxiety making many aspects of motherhood more challenging.

This current study aimed to gain insight into first-hand experiences of autistic mothers with sensory processing difficulties and to understand their role as mother navigating sensory processing challenges. The semi-structured interviews provided rich data about the sensory experiences of autistic mothers which was later analysed into 5 main themes which included; Antenatal, Sensory experiences in motherhood, Impact, Strategies and Needs and Diagnosis. The sensory experiences of the autistic mothers were similar in many areas resulting in a predominantly cohesive data set. All participants reported significant sensory over-reactivity within auditory and tactile sensory domains with some reports of sensory under-reactivity concerning proprioceptive and vestibular input during motherhood. Their sensory experiences were extreme, pervasive and impacted multiple facets of their mothering roles, including social and work roles and impacted the participants' mental health, fatigue levels and their ability to plan and organise their day to day routines. Several valuable strategies were employed by the participants to lessen the effects of their sensory processing difficulties including downtime, asking for support and linking with other autistic mothers.

This discussion will focus on the three key findings from this study; the sensory experiences of autistic mothers, strategies and the importance of diagnosis and lack of awareness.

### Sensory Processing Experiences in Motherhood

This current study identified the specific sensory difficulties autistic mothers experience. Other studies (Gardner et al., 2016; Rogers et al. 2016; Pohl et al., 2016, 2020) have referred to sensory experiences in the perinatal period of autistic mothers, however, did not expand upon them nor investigate post-natal sensory experiences.

All participants reported a significant increase in their sensory reactivity with auditory and tactile over-reactivity reported most frequently. Sensory over-reactivity impacted many aspects of their mothering role, including caregiving, social interaction, employment and organisational abilities resulting in higher than expected levels of stress, fatigue, overwhelm and anxiety. Studies have found sensory differences and over-reactivity are often a causal factor of increased stress and anxiety within the autistic population (Bundy et al. 2002; Cesaroni and Garber, 1991; Elwin et al., 2017; Landon et al., 2016; Lane et al., 2019; Marco et al., 2011; Milne et al., 2017; Milner et al., 2019; Smith & Sharp, 2013), which causes increased risk factors during pregnancy and later on during motherhood (Sundeline et al., 2018). All participants in this study reported behaviours consistent with higher levels of stress due to increased sensory over-reactivity, a finding that is consistent with earlier research.

Auditory over-reactivity was the most frequently reported sensory difficulty leading to stress, a finding that is consistent with previous research (Green & Ben-Sasson, 2010; Landon et al., 2016; Robertson & Simmons, 2015). Landon et al (2016) reported the most effective method to reduce sensory over-reactivity to sound was to withdraw from or limit exposure to it. However, this creates an immediate barrier for autistic mothers who, typically, being the prime caregiver for their child are by their role, exposed to constant auditory demands which are not always possible to withdraw from. The crying of their infant and later the incessant talking and use of noisy toys caused a significant impact. Although, some of the participants reported that as their children grew up, the sensory demand on their auditory system lessened as the teenage years drew in and their children were more solitary and less demanding, affording the parents much-needed downtime.

Most of the participants reported increased difficulty in tolerating tactile input. Once the baby was born, the constant need for physical comfort was difficult for all, a finding also reported by Mikkelsen et al (2018), who found autistic adults were more sensitive to tactile input when compared to neurotypical people. It is no surprise to find that breastfeeding was reported to be extremely painful and difficult to process for most mothers. Pohl et al. (2020) suggested that sensory difficulties experienced with breastfeeding may be unique to autistic mothers, yet interestingly participants in both studies (Pohl et al., 2020, and this study) always persisted to attempt breastfeeding despite experiencing high levels of pain and discomfort. Mothers, therefore, sacrificed their own needs in the best interests of their child, which was also reported in other studies (Gardner et al., 2016; Pohl 2020). This persistent tactile input from their infant is also noted to be a causal factor in being less able to participate in close relationships with their partners and other family members (Mikkelsen et al. 2018).

In addition, being unable to withdraw from the noxious sensory stimuli, autistic mothers were unable to reduce the demands on their sensory systems and make appropriate adaptive responses. This created a perception of the sensory input as threatening, causing a higher stress response. Smith and Sharp’s (2013) theory of the sensory avalanche, may help understand the participant's experiences. They assert that individuals can become more sensitized to sensory input if they perceive the input as threatening and cannot escape it, thus creating a vicious cycle leading to higher levels of sensory reactivity. This may also be the reason that some participants reported an adjusted lower threshold for tolerating sensory input following the birth of their child.

Two of the participants reported sensory under reactivity resulting in increased clumsiness, reduced body awareness and increased injury post-partum. This was a finding that was not previously reported in studies, therefore these results cast new light upon sensory under-reactivity responses within the sample.

A significant rate (6 out of 7 participants) of severe sickness lasting longer than 5 months gestation was experienced in the sample. According to the Royal College of Obstetricians and Gynaecology (Einarson et al., 2013), Hyperemesis Gravadium is a severe form of nausea and vomiting and has been found to affect about 0.3–3.6% of pregnant women. This means that within this small population sample, there was a high prevalence of prolonged and severe sickness, a finding that may be significant. Further research into this topic would, therefore, be indicated to ascertain if severe sickness in pregnancy could be a potential risk factor for autistic mothers linked to sensory processing difficulties.

For many of the participants, the sensory aspects of clinical environments were too stimulating, causing stress, yet simple environmental adaptations made a considerable difference, such as reducing the overhead lighting and avoiding open plan spaces to contain noise levels. In addition to staff training, architects and designers need to consider the sensory impacts of design upon its service users and where possible, ensure design decisions are informed by the sensory needs of this population. The Sensory Design Matrix Tool, an architectural and design option generator, whereby the service users sensory needs are matched to potential design and architectural options (Mostafa, 2008), has been created, however up until now, the sensory needs of this population were not known.

### Strategies

All of the participants cited downtime and time alone as one of the most important restorative factors in managing their sensory overwhelm. However, for most of the participants, it was very difficult to achieve this when they became a mother. Instead, they sought to avoid or withdraw from social and work commitments as a means to gain more downtime, however, reported that at times this would lead to isolation which was linked to low mood, both findings were echoed in Smith and Sharp’s (2013) and Landon et al’s (2016) research. When withdrawing or seeking solace, participants reported that creating a calming space was vital to provide a soothing sensory experience. This often included low lighting, calming music, soft furnishings and using methods to block out or dampen external sensory stimuli.

Very little research offers specific advice to autistic mothers about sensory adaptations and affordances to dampen down sensory stimuli. When the participants were faced with a lack of relatable literature, most turned to the internet to find out if other autistic mothers felt as they did, often to be met with a group of like-minded people with whom they found solace and received validation for their experiences without fear of judgement. Accessing online autistic communities was for some, a lifeline that led to real-life friendships. This finding mirrors recent research by Leedman et al. (2020) who found when autistic women found connections with other autistic women, this provided relatable information and autonomy which helped them make better sense of their lives.

It is important to note that despite all of the participants in this study experiencing marked sensory processing challenges, they were able to act in the best interests of their child and frequently reported circumstances where they placed the needs of their child over and above their own, yet, they were often concerned they would encounter stigma from health care professionals.

### Diagnosis and Lack of Awareness

The results of this study build upon existing evidence which has found women are often delayed in receiving a timely diagnosis, resulting in a lack of appropriate information and support to understand their unique sensory experiences. This finding mirrors Gardner et al’s (2016) low rates of diagnosed autistic mothers within their sample. Receiving a diagnosis following the birth of their child created increased barriers as participants experienced heightened sensory reactivity during pregnancy and beyond, yet were unable to understand the reasons why (Gardner et al., 2016; Rogers et al., 2016). It is reasonable to propose that the demands of motherhood, coupled with the increase in sensory stimuli and the inability to employ previous coping strategies led to sensory-overstimulation and increased stress. Furthermore, the study by Pohl et al (2020) found that of the 410 autistic mothers studied, 60% of them were undiagnosed until after the birth of their child.

Additionally, Leedham et al (2020) and Pohl et al. (2016) found medical professionals did not adequately understand the needs of autistic mothers nor were they able to respond appropriately to them, with participants requests for small adaptations to reduce the impact of their sensory overwhelm often being dismissed. Furthermore, autistic mothers experienced increased difficulties communicating with professionals yet most autistic mothers wanted more support. It is important for “the right” person to help an autistic person, or they can make matters worse and increase stress levels (Smith and Sharp, 2013) therefore, accessing health care professionals who have a firm understanding of autism and postgraduate training in sensory integration and sensory processing difficulties in motherhood is essential.

There is a clear gap in current diagnostic processes which impacts on autistic women. Potential autistic mothers should receive a timely diagnosis in addition to having suitably trained skilled clinicians to provide sensory advice, support and coaching to newly diagnosed mothers. This can then serve as a protective factor to reduce risks to autistic mothers as they embark on their journey into motherhood.

### Strengths and Study Limitations

To the researcher’s knowledge, this is the first study to specifically explore the sensory experiences of autistic mothers, therefore it adds new insight into this field of enquiry. Adopting a qualitative method enabled the researcher to gather rich data about this subject, where the central issues were able to be explored. Being an insider researcher enabled the researcher to understand the core issues being explored from a lived perspective and as a result of her own experiences and training, was able to ask additional insightful probing questions to investigate the participants experiences further. The researcher’s insider status was revealed to the participants before the interviews taking place; this lead to many of the participants expressing they felt the researcher truly “understood” them. This may have resulted in the participants sharing more openly about their experiences. Diligent methods were employed throughout the study to reduce bias and increase trustworthiness and rigour. The researcher ensured she was sensitive to represent participants experiences authentically.

The researcher made every effort to involve autistic people in all stages of the research process which was a strength, a view supported by Gillespie-Lynch (2017), who asserts involving autistic people as empowered collaborators in the research process will lead to more accurate understandings of autism and reduce stigma. The findings provide a foundation for future research to establish appropriate ways to support autistic mothers, however, with a small sample, the results of this study are not generalisable to all autistic mothers.

The sample was exclusively recruited via the Cambridge Autism Research Database (CARD), and all participants were living in the UK, white, middle-aged and able to communicate well verbally. They did not report having any learning or severe mental health difficulties, as such this sample may not be truly representative of all autistic mothers and other geographical, socio-economical areas. Most of the participants (5 out of 7) had children with a diagnosis of autism, it was not within the scope of this study to explore if their sensory experiences were greater or lesser when compared to the mothers with children without a diagnosis, however, this may have added to the mother's sensory experiences.

Since sensory functioning in autism is still an evolving theory and field of enquiry, it is possible that the participants were not fully aware of the full scope of their sensory experiences and how this may have affected them, as a result, they may have been unable to provide an informed account of the multiple areas of sensory processing including praxis, posture and discrimination. The researcher did provide a brief introduction at the start of the interview; however, this may not have been enough to gain rich data regarding all sensory systems. A mixed-methods approach using a sensory assessment tool alongside semi-structured interviews would have been helpful to better inform the researcher about each participant’s sensory profile.

### Broader Clinical Implications

Professionals encountering autistic mothers need to develop a deeper understanding of the sensory needs of this population so that autistic mothers are identified earlier and receive tailored and skilful sensory strategies to reduce the impact of stress and anxiety. Professionals, local service providers and employers need to receive training and awareness about the sensory needs of this population so that policy and models of service delivery can be developed to improve support mechanisms that are tailored to the needs of this population. An Occupational Therapist with post-graduate training in Ayres Sensory Integration would be a suitably skilled professional to support autistic mothers by conducting comprehensive assessments to support the development of tailored sensory strategies and to apply graded activity analysis to modify the task or environment, highlighting a possible emerging role. Autism is a lifelong neurodevelopmental condition and is covered under the Disability Discrimination Act (2010) and the Equality Act (2010), meaning that services must be seen to be making reasonable adjustments to enable autistic people to access services without discrimination and with reduced barriers. The challenges autistic mothers reported due to their sensory processing difficulties significantly affected their engagement and participation in meaningful daily occupations and as such would benefit from receiving a Health and Social Care assessment to see if they may be eligible for support.

For some autistic mothers, for instance, receiving access to Direct Payments to purchase helpful sensory equipment, or to hire a cleaner or home-based support worker when they feel overwhelmed may be beneficial in supporting access to downtime and offer respite. Existing local initiatives to support parents with disabilities could be extended or appropriated to include autistic mothers. However, due to the high demand for services and low availability of resources, stakeholders and policy writers may need to reframe the current thresholds for accessing such support as autistic mothers may not currently meet the thresholds.

### Future Research

The findings within this study, and many others like it, highlight the urgency for the development of additional research to investigate appropriate diagnostic tools and procedures that can identify autistic women earlier and improve their trajectory. Literature relating to autistic mothers has identified higher risk factors relating to stress and sensory processing, and it would be important for further research to focus on and identify causal factors, for example, environmental factors and sensory processing difficulties. Future research should also investigate the broader impacts of sensory processing including mental health, social and employment implications. It is hoped that this study will offer a foundation for further research and inform service and policy development on how to make small adaptations to improve accessibility.

## Appendix 1: Interview Guide

### Researcher Interview Schedule

Interview questions

- Researcher welcomes the participant to the interview
  o Thank you for participating in my research and for giving your time to this project.
- Researcher introduces herself to the participant –
  o I am a qualified Occupational Therapist and work with individuals with ASC. I am currently carrying out my MSc research project and am in the final year of my MSc at Ulster University. My research project aims to explore the sensory experiences of mothers with ASC in the hope that it will raise awareness and help to inform service provision.
  o This interview is recorded with your permission and will be transcribed afterwards. Your personal details will remain anonymous.
  o There are no right or wrong answers, I am interested in your own personal experiences and how they have impacted your life.
  o The interview is confidential, however, If you disclose any information which indicates that either you or someone you know is at risk of harm, I must break confidentiality and inform my supervisors and the University, if this happens, I will inform you that I am required to follow this process.

Warm up questions

1. Were you aware of any sensory experiences before you had your child/ children?
  - What were your sensory experiences like growing up?
  - What were your sensory experiences like as an adult?
  - Pre-pregnancy?
  - Did you have any sensory experiences before your baby?
2. Can you describe what your sensory experiences were like after you had your child/ children?
  - Did pregnancy alter your sensory experiences?
  - What were your sensory experiences like during labour and birth?
  - What were your sensory experiences like in the early years of your baby’s life?
    i. Toddler years
  - What were your sensory experiences like during the teen years of your child’s life?

Main questions

3. Do you feel your sensory experiences have influenced or impacted your role as a mother in any way?
  - Did anything surprise you at all/ unexpected?
  - Self-caring and caring for a child?
  - Social interaction
  - Leisure
  - Occupation?
  - Relationships?
4. Can you describe how your sensory experiences impact the things you do each day?
  - Personal care – Washing/ bathing/ grooming/ dressing/ feeding/ drinking
  - Productivity – Work/ homemaking/ cooking/ cleaning/ mothering
  - Leisure – Socialising/ fitness/ outings
  - Relationships?
5. What coping strategies, if any, do you employ or find helpful?
  - How have you managed so far?
  - Informal support?
  - Formal support?
  - Internal resources?
  - What works?
  - What hasn’t helped?

Closing question:

6. Do you have any advice you would give to others?
  - Any services that have helped?
  - How did they make a difference?
  - What are your top tips for managing sensory experiences?
  - Is there was one thing that you found very helpful that you think others may find helpful too?
  - What changes would be helpful for you to help you manage your sensory needs?
  - Anything else you would like to add?
• Researcher thanks the participant for attending and reiterate that if anything they discussed made them feel unsettled, or upset, they will be provided with further details of organisations that they can contact should they wish to.
• Researcher refers to the protocol for dealing with distress or upset.
• Remind the participant that they will receive a transcript of their interview via email (password protected) as a record of their interview.
• Researcher sends the participant the debriefing form.
